# Effect of Different Sports Practice on Sleep Quality and Quality of Life in Children and Adolescents: Randomized Clinical Trial

**DOI:** 10.1186/s40798-021-00376-w

**Published:** 2021-11-17

**Authors:** Camila Cassemiro Rosa, William Rodrigues Tebar, Crystian Bittencourt Soares Oliveira, Breno Quintella Farah, Juliano Casonatto, Bruna Thamyres Ciccotti Saraiva, Diego Giulliano Destro Christofaro

**Affiliations:** 1 Department of Physical Education, School of Technology and Sciences, São Paulo State University (UNESP), Roberto Simonsen Street, n° 305, Presidente Prudente, São Paulo 19060-900 Brazil; 2 Universidade do Oeste Paulista (UNOESTE), Presidente Prudente, SP Brazil; 3 Universidade Federal Rural de Pernambuco, Recife, PE Brazil; 4 Universidade do Norte do Paraná (UNOPAR), Londrina, PR Brazil

**Keywords:** Physical activity, Motor behavior, Children and adolescents, Sleep quality, Quality of life, Sport

## Abstract

**Background:**

Sports practice can promote several health benefits in pediatric populations; nonetheless, most of the studies that investigated these benefits are of cross-sectional design. Thus, our objective was to verify the effectiveness of two types of physical activities through sports, judo and ball games (soccer, volleyball, handball, and basketball) on the quality of sleep and life of Brazilian children and adolescents.

**Methods:**

The study is a randomized clinical trial, conducted with 65 participants of both sexes (6–15 years old) in a philanthropic institution in Brazil. The variables investigated were the quality of sleep and life, using the mini-sleep questionnaire and KidsCreen-52 questionnaires (this instrument has a scale ranging from 0 to 100, where 100 is the best value for each domain), respectively. The interventions carried out during 12 weeks (twice a week) were judo and ball games. In the statistical analysis, analysis of variance (ANOVA) for repeated measures was used and the level of statistical significance used was 5%.

**Results:**

Judo (*P* = 0.032) and ball games (*P* = 0.005) contributed to improving the quality of sleep in the participants. Considering the score of the domains of quality of life, judo and ball games significantly increased the perception of health and physical activity [mean = 6.9 (8.3%) and 8.91 (12.2%) points, respectively], autonomy [mean = 5.81 (7.3%) and 5.00 (6.9%) points], friends and social support (mean = 2.83 (3.8%) and 12.00 (15.9%) points), provocation and bullying [mean = 10.21 (18.1%) and 2.14 (4.1%) points].

**Conclusion:**

It is concluded that both judo and ball games brought benefits to the quality of sleep and life of children and adolescents. Health promotion actions should encourage the increase in sports practice in children and adolescents to improve sleep and quality of life.

## Key Points

- Few randomized clinical trials have been carried out with the aim of verifying possible benefits of different sports practices on the quality of sleep and life in pediatric populations.
- We observed that both sports modalities (judo and ball games) improved the quality of sleep in children and adolescents participating in this study.
- The results of this study may contribute to the development of health promotion strategies for young people, considering their well-being through the encouragement of sports practice.

## Background

Childhood and adolescence are important periods of life for the development of healthy lifestyle habits and important physiological processes that can be carried into adult life [1]. Among these physiological processes is sleep quality, as it is at this stage that major changes occur in the neuromotor and hormonal systems, in which physical rest during sleep is extremely important for child growth and development, as well as changes and physical maturation in adolescents [2]. Therefore, sleep is characterized by a physiological need that influences biological functions, such as organic restoration, energy conservation, and physical and mental balance and when there is an absence or sleep disorders, neurodegenerative diseases develop, interfering in the individual's quality of life [3].

Poor sleep quality can be associated with several problems in children and adolescents, such as fatigue, anxiety, and depression, and can also interfere in the learning and academic performance of these young people [4]. Furthermore, quality of life also tends to be affected, since poor sleep can affect children and adolescents' self-perception of health and social relationships [5]. However, one health promotion action that is low cost and easy to apply and that can be used to improve the quality of sleep and life of children and adolescents is physical activity. In a study conducted with adolescents, it was observed that the quantity of moderate physical activity was associated with better sleep quality [6]. Considering quality of life, [7] a study with 276 Norwegian children observed that physical activity was associated with physical and mental well-being. Another study found that moderate- or high-frequency sports were associated with better quality of life in adolescents [8]. Additionally, high levels of physical exercise, low levels of sedentary behavior, and enough sleep have all been linked to improved mental health in children and adolescents [9–11]. The fact that physical activity, sedentary time, and sleep have been considered individually is worrisome, because research has demonstrated that these three behaviors are interdependent and should be considered together [12, 13].

In this sense, considering different sports among Brazilian children and adolescents, judo is a very popular sport, which has been associated with increases in other health parameters, such as bone mineral density and cardiac autonomic modulation [14, 15]. Due to its characteristics of discipline and promoting greater social interaction, in addition to high energy expenditure and physical conditioning, judo could be related to longer lasting, quality sleep, resulting in greater well-being. Ball sports, which are common in Physical Education classes in Brazilian schools, have been associated with a better body profile. In addition, the effect of ball sports on the quality of sleep and life of children and adolescents has been reported in other countries [16].

In particular, no study, to our knowledge, has applied a longitudinal design in this field. The majority of studies that investigated the relationship between the practice of physical activity and quality of sleep and life in children and adolescents are cross-sectional [17, 18]. Another factor to be considered is that adjustments in possible confounding variables such as sex, age, and somatic maturation should be considered as potential confounders. Finally, most intervention studies aiming to verify the influences of physical activity on the quality of sleep and life were carried out with adults from developed countries [19, 20].

Therefore, the objective of the present study was to verify the effectiveness of two types of physical activities through sports, judo and ball games (soccer, volleyball, handball, and basketball) in the quality of sleep and life of Brazilian children and adolescents.

## Methods

### Sample and Design

The present study is a randomized Clinical Trial (Registered in the Clinical Trials platform: NCT03068000), carried out in a philanthropic institution in the city of Presidente Prudente (located in the southeastern region of Brazil). First, there was a meeting with the direction of the philanthropic institution explaining the types of sports practices to be offered to children and adolescents, as well as the evaluations to be carried out and the randomization process. Later, a meeting was held with parents and children and adolescents who were enrolled in this philanthropic institution, explaining that two types of sports practice would be offered, judo and ball games, and the evaluations of the health parameters that would be offered. However, the need for randomization was explained, as it is a scientific project. Both the management and the parents and the children and adolescents agreed with the proposal, and the sports activities offered were included in the activities of the philanthropic institution.

The sample consisted of 85 participants of both sexes, randomized into two groups (42 in judo and 43 in ball games). Sample size calculation was based on a previous study that evaluated the effects of sports on cardiac autonomic modulation (by heart rate variability) in children and adolescents [15]. Therefore, a standard deviation of 4 was used, and sampling power of 80% and significance of 5% were used. The minimum sample size was 62 participants. At the end of the study, 63 participants completed all assessments (judo = 29 and ball games = 36). This study was approved by the Research Ethics Committee of Universidade Estadual Paulista (CAAE: 26702414.0.0000.5402).

### Inclusion and Exclusion Criteria

As inclusion criteria, participants were required to be regularly enrolled in the philanthropic institution where the study was conducted, be between 6 and 15 years of age, and present a free and informed consent form signed by parents and/or guardians. As exclusion criteria, participants could not be taking any type of medication that would influence the variables evaluated, present any type of orthopedic problem that would prevent the performance of activities, and not be pregnant.

### Sample Randomization Process

After data collection at the baseline, the young people who participated in this study were randomly distributed into one of two groups: systematic practice of judo or sports through ball games. The randomization process was carried out by a researcher who was not part of this project (with the objective of blinding the sample allocation process) through a sequence of numbers generated on the website http://www.randomization.com.

**Fig. 1:**
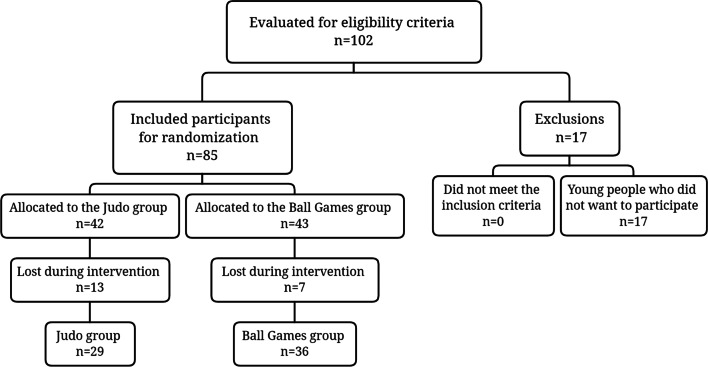
Flowchart of the study sample

The sample allocation process was performed secretly with opaque envelopes, sealed and following the numerical sequence of the allocation. In the first session of sports practices, the envelope was opened in front of the participant, informing them which group they belonged to. This whole process is in line with CONSORT recommendations for randomized clinical trials [21]. The sample distribution flowchart is shown in Fig. 1.

### Sports Practice Protocol

#### Judo

Judo interventions were performed twice a week, lasting 60 min per session. The practice of judo was divided into general exercises, beginning with a warm-up and stretching specific to the sport, which lasted approximately 10 min. After the warm-up, specific exercises for this type of martial art were performed, such as shock absorption, fall cushioning (Ukemi-waza), immobilization techniques (hon-kesa-gatame and tate-shiho-gatame), and projection (o-soto-gari, o-goshi, ashi-guruma, koshi-guruma, tai-otoshi, and others). At the end of the session, a combat simulation (randori) was carried out and a return to calm with relaxation activities.

### Ball Games

The alternative practice to judo was called ball games. This type of sports practice consisted of sports which used a ball, including football, volleyball, basketball, and handball. These sports were included because they are common in Physical Education classes in Brazilian schools. The ball games were held twice a week, lasting 60 min per session. On each day of the week, a type of ball sport was performed (example: 1st week of the month: 1st day of the week: football; 2nd day of the week: volleyball; 2nd week of the month: 1st day of the week: handball; and 2nd day of the week: basketball), divided into general exercises, beginning with a warm-up and specific stretching with a ball, for approximately ten minutes. Subsequently, specific exercises of the modality were performed: fundamentals of sport with ball, exercises with ball. At the end of each session, specific games were given for each modality performed in that session, followed by a return to calm with relaxation exercises.

### Application of Questionnaires

As the sample of the present study consisted of children aged 6 years and over and adolescents aged up to 15 years, some precautions were taken for the application of the questionnaires. In a classroom provided by the philanthropic institution where the study was carried out, three researchers stayed to apply the questionnaire to a maximum of six children. In case there was any difficulty in understanding the questionnaire by the child, the assessment was carried out face to face.

### Sleep Quality

Sleep quality was assessed using the “Mini-Sleep Questionnaire” developed by Zomer et al. [22], which evaluates aspects related to sleep pattern, as well as the frequency with which these aspects can occur. The total score of the scale ranges from 0 to 60, being, respectively: (0–9 points) very good sleep; (10–24) good sleep; (25–27) slightly altered sleep; (28–30) moderately altered sleep; and (over 30) very altered sleep. This instrument has been validated for the Brazilian population [23] and has good reproducibility indices [24].

### Quality of Life

Quality of life was assessed using the KIDSCREEN-52 questionnaire. This instrument was developed in European countries to assess the quality of life in young people [25]. The questionnaire consists of 52 questions that are directed to the perception of ten dimensions related to quality of life.

The answers to the questions are attributed on a scale similar to Likert, which varies from one to five points, to assess and identify the frequency of the behavior/feelings, or the intensity of specific attitudes, in the pre- and post-intervention evaluation. The score of each dimension was computed, using a syntax that considers the answers of each group of questions that compose the dimension, with the questions being weighted equally. In the dimensions, the equivalent final scores are recorded on a measurement scale, between 0 and 100, where 0 is the lowest and 100 the highest in relation to the perception of quality of life. This instrument was translated and validated for use in Brazilian children and adolescents [26].

### Somatic Maturation

Somatic maturation was used to assess biological development, through the Peak of Growth Velocity (PGV) analysis [27], which uses anthropometric measures of weight, height, and trunk-head height. The formula for females is: − *9.376* + *0.0001882 *×* (TL *×* TC)* + *0.0022 *×* (I *×* TL)* + *0.005841 *×* (I *× *TL)* − *0.002658 *×* (I *×* W)* + *0.07693 *×* (W/H)*, where TL corresponds to trunk length, TC to trunk-cephalic height, and W (weight) and H (height). In males, the formula is: − *9.236* + *0.0002708 *×* (TL *×* TC) – 0.001663 *×* (I *×* TL)* + *0.007216 *×* (I *×* TC)* + *0.02292 *×* (W/H)*.

### Statistics Analysis

In the statistical analysis, analysis of variance (ANOVA) was used for repeated measures in which the possible differences between the groups were determined. Precision measures for differences between groups were also adopted by presenting the mean and standard deviation values, measures determined by CONSORT to be used in studies of randomized clinical trials. An intention-to-treat analysis was also carried out in which an attempt was made to evaluate all participants at baseline and after the intervention, regardless of their number of absences during the training session, thereby preserving the benefit of the process of randomization [28]. Sex, age, and peak growth velocity were used as adjustment variables for comparison of post- vs. pre-differences according to group (judo vs. ball games). Effect sizes were calculated to estimate the magnitude of differences between groups [29]. The level of statistical significance adopted was 5%. The statistical software used was SPSS (version 15.0).

## Results

Eighty-five children/adolescents were randomized, 42 to the judo group and 43 to the ball games group. Of the 85 initially randomized participants, 20 did not complete all sessions or evaluations and were considered to be sample losses (23.5%). Table 1 shows the characteristics of the sample at the baseline moment. No statistically significant differences were observed at baseline in any of the variables investigated.

**Table 1:** Characteristic of the sample at baseline

Variables	Judo (*n* = 42)	Ball games (*n* = 43)	*P*
	Male (*n* = 22)	Male (*n* = 30)	
	Female (*n* = 20)	Female (*n* = 13)	

	Mean (SD)	Mean (SD)	
Age (years)	9.90 (1.56)	9.96 (1.51)	0.885
Weight (kg)	38.81 (14.57)	43.55 (19.60)	0.239
Height (cm)	136.57 (27.01)	141.97 (13.40)	0.278
PGV	− 3.44 (1.03)	− 3.60 (1.42)	0.655
Sleep quality	24.71 (9.20)	27.02 (10.21)	0.306
*Dimensions of quality of life*			
Health and Physical Activity	72.07 (25.53)	65.30 (22.84)	0.243
Feelings	76.67 (21.49)	78.79 (26.06)	0.639
Emotional state	45.79 (25.99)	52.00 (15.99)	0.263
Self-perception	48.84 (23.66)	50.57 (26.43)	0.720
Autonomy	71.02 (27.53)	67.05 (19.69)	0.487
Family Environment	78.09 (21.83)	78.80 (18.58)	0.881
Financial Aspect	55.48 (29.71)	47.68 (26.54)	0.239
Friends and Social Support	70.37 (26.98)	64.89 (24.73)	0.384
School environment	73.98 (27.11)	69.27 (26.21)	0.437
Provocation / Bullying	43.80 (32.92)	51.57 (31.10)	0.294

*PGV* peak of growth velocity. The score for each quality of life domain ranges from 0 to 100

**Fig. 2:**
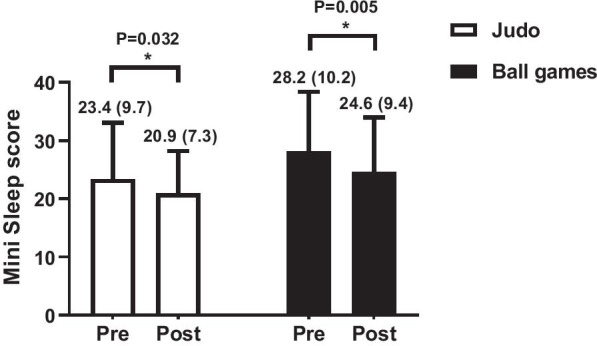
Effect of 12-week intervention with judo or ball games training on the sleep quality of children and adolescents. *Significant difference between pre versus post within groups. Mini-sleep score = values expressed as mean and standard deviation. (The higher the score, the worse the sleep quality.) Effect size between groups was 0.17 and power of 0.70, with a significant main effect for time (*P* = 0.007) and non-significant group by time interaction (*P* = 0.612)

Figure 2 presents information on the effectiveness of training in the quality of sleep of children and adolescents participating in the project. Significance main effect was observed for time (*P* = 0.007), while there was no significant group by time interaction (*P* = 0.612). Significant decreases of 2.5 points (*P* = 0.032) and 3.6 points (*P* = 0.005) in the sleep quality scores for judo and ball games between pre–post-assessments, respectively, demonstrated the benefits of both interventions, since on the scale of the mini-sleep questionnaire, the higher the score, the worse the quality of sleep. Effect size between groups was 0.17 and power of 0.70. Furthermore, the marginally non-significant main effect for group (*P* = 0.071) supports a trend for a moderately better sleep quality in the judo group (good sleep) compared with ball games (altered sleep) across the whole study period, but with a relatively low effect size.

Table 2 presents information on the effects of three months of training in judo and ball games on the quality of life of children and adolescents. There was a significant improvement in the domain of health and physical activity (power 0.31) over time in both types of training, with 8.3% of increase in judo and 12.2% of increase in ball games group. No significant differences were observed in the quality of life domain of feelings (power 0.05), emotional state (power 0.98), family environment (power 0.17), financial aspects (power 0.41), and school environment (power 0.99) in any of the groups after the intervention. A significant interaction between group and time (*P* = 0.018) was observed in the self-perception of children and adolescents (power 0.99), where those who trained ball games decreased the score in 12.7%, while those who trained judo increased the same score in 1.1% (*P* = 0.009 for post- vs. pre-difference comparison between group). Considering the autonomy domain (power 0.07), there was improvement in both the judo group (7.3%) and the ball games group (6.9%). In the domain of friends and social support (power 0.99), both training types contributed to improving quality of life, with increases of 3.8% in the judo group and 15.9% in the ball games group. Finally, there were also significant improvements in the domain of provocation/bullying (power 0.99) after the interventions, by increasing 18.1% in judo group and 4.1% in ball games group.

**Table 2:** Effect of 12-week intervention with two types of training on the quality of life of children and adolescents

	Judo	Difference	Ball games	Difference	Effect	*P*
	(*n* = 29)	Post versus Pre	(*n* = 36)	Post versus Pre		
Health and physical activity					Group	0.061
Pre-intervention	76.44 (4.38)	6.89 (3.40)	63.87 (4.23)	8.91 (3.72)	Time	**0.002**
Post-12-week intervention	83.33 (3.48)	8.3%	72.78 (3.27)	12.2%	Group × Time	0.688
*Effect size between groups*	*0.10*					
Feelings					Group	0.179
Pre-intervention	79.13 (4.21)	0.18 (3.68)	77.90 (2.90)	0.22 (3.49)	Time	0.453
Post-12-week intervention	79.31 (4.18)	0.2%	78.12 (2.62)	0.3%	Group × Time	0.190
*Effect size between groups*	*0.01*					
Emotional state					Group	0.671
Pre-intervention	48.14 (5.17)	3.04 (2.95)	50.46 (2.91)	− 2.19 (3.41)	Time	0.457
Post-12-week intervention	51.19 (5.62)	5.9%	48.27 (4.53)	− 4.3%	Group × Time	0.080
*Effect size between groups*	*0.29*					
Self-perception					Group	0.686
Pre-intervention	48.10 (4.31)	0.52 (4.71)*	50.59 (2.86)	− 6.47 (2.79)*	Time	0.594
Post-12-week intervention	48.61 (3.42)	1.1%	44.12 (2.32)	− 12.7%	Group × Time	**0.018**
*Effect size between groups*	*0.34*					
Autonomy					Group	**0.003**
Pre-intervention	73.39 (4.85)	5.81 (4.24)	66.91 (3.37)	5.00 (4.23)	Time	**0.005**
Post-12-week intervention	79.19 (4.00)	7.3%	71.91 (3.32)	6.9%	Group × Time	0.376
*Effect size between groups*	*0.03*					
Family Environment					Group	0.328
Pre-intervention	81.40 (4.06)	3.57 (2.60)	77.97 (3.09)	2.38 (3.29)	Time	0.332
Post-12-week intervention	84.97 (3.97)	4.2%	80.36 (3.27)	2.9%	Group × Time	0.597
*Effect size between groups*	*0.07*					
Financial Aspect					Group	0.331
Pre-intervention	57.78 (5.40)	0.27 (6.05)	47.22 (4.29)	4.39 (5.82)	Time	0.313
Post-12-week intervention	58.05 (5.19)	0.4%	51.62 (4.59)	8,5%	Group × Time	0.196
*Effect size between groups*	*0.12*					
Friends and Social Support					Group	0.202
Pre-intervention	71.00 (5.19)	2.83 (5.04)	63.89 (4.50)	12.08 (5.64)	Time	**0.029**
Post-12-week intervention	73.81 (4.70)	3.8%	75.97 (3.67)	15.9%	Group × Time	0.692
*Effect size between groups*	*0.30*					
School environment					Group	0.179
Pre-intervention	81.32 (3.87)	− 7.33 (3.71)	70.18 (3.95)	0.13 (3.72)	Time	0.410
Post-12-week intervention	73.99 (4.54)	− 9.0%	70.31 (3.61)	0.2%	Group × Time	0.696
*Effect size between groups*	*0.36*					
Provocation/Bullying					Group	0.972
Pre-intervention	46.00 (6.41)	10.21 (3.84)	50.24 (5.08)	2.14 (2.71)	Time	**0.009**
Post-12-week intervention	56.18 (5.75)	18.1%	52.38 (5.34)	4.1%	Group × Time	0.262
*Effect size between groups*	*0.45*					

Bold values indicate statistically significant results (*P* < 0.05)

*Statistical significance in analysis of variance adjusted for sex, age, and somatic maturation for comparison of post- versus pre-difference between groups

## Discussion

The main findings of the present study showed that the practice of different sports (judo and ball games) for 12 weeks was effective in improving the sleep quality of the children and adolescents. Both sports practices were also effective in improving some quality of life domains such as physical activity and health, autonomy, social interaction with friends, and provocation and bullying.

In our study, both types of sports collaborated to improve sleep quality over a 12-week period, with no interaction between the groups, although the effect size was small. A study [30] with German adolescents also observed that leisure sports were associated with better sleep quality in this population. One of the possible reasons for this improvement can be derived from the benefits of sports practice, including changes in neurotransmitters and biochemical processes that could be associated with better sleep quality [31]. Another factor is that sports practice in adolescence was associated with better mental health [32], which could successively contribute to the quality of sleep, since better mental health has been associated with better sleep quality in children and adolescents [33, 34].

Another important finding of our study was to verify the influence of judo and ball games on the quality of life of children and adolescents participating in the intervention. Judo and ball games (composed of ball sports, soccer, volleyball, handball, and basketball) were chosen because they are a good reflection of popular Brazilian sports. Judo is an Olympic sport with a long tradition in Brazil, and team sports with a ball are the most commonly played games in Physical Education classes in Brazilian schools. Other studies observed positive results in the quality of life after the intervention through sports practice. Aguilar-Cordero et al. [35], in a clinical trial with obese Spanish children, observed improvements in physical and mental components related to quality of life (using the SF-10 questionnaire). A 22-week intervention in aerobic and resistance training groups of adolescents showed significant increase in quality of life when compared to controls, being quality of life assessed by Pediatric Quality of Life questionnaire [36]. Another intervention using concentric and eccentric cycling training program in obese adolescents showed significant increases in the health-related quality of life scores in both groups after a 12-week period of training, assessed by The Vécu et Santé Percue de l’Adolescent (VSP-A) questionnaire [37]. However, the different modalities, period of interventions, and instruments assessing quality of life precluded broader comparisons with the present study.

In judo, we highlight the positive effects in the domains of self-perception, in which the young people participating in this study presented self-knowledge about issues related to their own personal satisfaction and in relation to their friends and colleagues, and autonomy in which they pointed out the frequency with which they were able to have free time to carry out their activities and freedom to choose what they would do in their free time. One of the reasons for the observed results is perhaps motivated by the characteristic of this sport. Judo is a martial art modality originating in the East, which works on philosophical principles, discipline, self-control, and mental and physical balance [38]. This could contribute to a better perception of young people about themselves, as well helping them to be more autonomous in their choices. On the other hand, ball games could develop greater socialization due to their collective aspect, also contributing to the development of autonomy. This fact may also explain the improvements in timing for the “provocation/bullying” dimension in favor of Judo.

In both modalities, significant increases were observed in the score related to physical activity and health. This dimension measures how children and adolescents consider their physical health to be and their conditions to perform daily activities. A randomized, cluster-controlled clinical trial conducted with school children in Switzerland [39] identified that after three years of a multi-component physical activity program, including sports, the young people showed improvement in aerobic fitness and motor coordination, with increased scores for quality of life mainly in the health domain. Complementing to these findings, another study reported that better physical fitness was associated with a greater perception of health in children and adolescents [40].

Judo and ball games were also related to better social interaction with friends, considering the domain of friends and social support. This dimension quantifies how often the participants are able to be in the presence of friends and how safe they feel to talk about private matters with them. Improvements were observed in the score of this domain over the three months in both judo and ball games. Similar results were observed by Urzeala et al. [41] in a study with Romanian children with type 1 diabetes who underwent an interdisciplinary health intervention, which included sports. The authors found improvements in social relationships and socializing among friends. Sports practice tends to strengthen interpersonal relationships, as it can promote greater interactions and sharing of ideas among children and adolescents.

Regarding the provocation and bullying dimension, an improvement was observed in the score of both groups. However, this was more evident in children and adolescents who practiced judo; that is, children and adolescents who were threatened or were victims of some discriminatory situation realized that, after the intervention, they felt safer and more confident about negative attitudes from other people [42]. A study investigating more than four thousand Italian adolescents observed that the practice of sports was an ally against bullying, with a reduction in these situations in adolescents who practiced sports and who were victims of bullying [43]. A study with 283 high school students observed that the practices of martial arts promoted benefits in relation to bullying when compared to the control group. The martial arts, especially those of oriental origin include respect, loyalty, and solidarity in their principles [44] and such factors could be responsible for the improvement in this domain of quality of life in the sample of the present study. In ball games, we emphasize that team sports can develop solidarity aspects among the participants, with respect to being part of a team [45].

Considering the dimensions: feelings, which reports and quantifies the intensity with which they feel “important” and satisfied with life, as well as the emotional state, which measures the frequency with which mood changes occur; family environment, which also verifies the frequency of the type of relationship and trust with parents; financial aspects that quantify the financial and economic conditions to carry out activities or buy something they want, and finally; a school environment which measures whether the participants were satisfied with the school environment and their performance, in the present study no significant results were found. One of the hypotheses is that the results are dependent on these environments; therefore, the family context and the economic conditions in which they are inserted, have a greater influence on the lives of these children and adolescents than the sport itself.

Among the limitations to be considered in this study is the sample loss greater than 15%, considered as one of the qualitative aspects of clinical trial studies. However, we emphasize that the study was carried out in partnership with a philanthropic institution that serves children and adolescents from the periphery and with low socioeconomic conditions, who often have difficulty participating assiduously in the activities. Another factor was the absence of a control group that did not practice sports for comparison purposes with the sports in this study. The sample size calculation was based on a previous study by our group that had cardiovascular indicators as an outcome, which may have affected the power of the analyses. Finally, the absence of assessment of physical activity outside the practice of sports offered in the philanthropic institution and sedentary behavior are possible variables that could influence the results and should be considered as a limitation. Despite this limitation, we emphasize the possible benefits evidenced in the quality of sleep and life in children and adolescents with low economic conditions. Another positive aspect is the randomization of the sample and the longitudinal design of the study, making it possible to verify the cause and effect relationship.

With regard to the practical applicability of this study, although the effect size in most of the indicators was small, we observed that the practice of sports in different modalities, whether collective modalities with a ball or martial arts modalities, is essential for quality of sleep and life for children and adolescents. In this sense, health promotion actions aimed at increasing the quality of sleep and life of children and adolescents must prioritize incentives to practice sports, especially considering several government programs (whether carried out in schools, philanthropic institutions, or public squares).

## Conclusions

In summary, judo and ball games were effective in improving the quality of sleep and some life domains of children and adolescents. Health promotion actions for children and adolescents should aim to increase sports practice by young people.

## Data Availability

Data from this study may be available from the corresponding author on reasonable request.

## Abbreviations

ANOVA: Analysis of variance
PVC: Peak of growth velocity
TL: Trunk length
TCH: Trunk-cephalic height
